# Buckwheat: a crop from outside the major Chinese domestication centres? A review of the archaeobotanical, palynological and genetic evidence

**DOI:** 10.1007/s00334-017-0649-4

**Published:** 2017-12-02

**Authors:** Harriet V. Hunt, Xue Shang, Martin K. Jones

**Affiliations:** 1 McDonald Institute for Archaeological Research, University of Cambridge, Downing Street, Cambridge, CB2 3ER UK; 2 Department of Archaeology and Anthropology, University of Chinese Academy of Sciences, 19A, Yuquan Road, Beijing, 100049 China; 3 Division of Archaeology, University of Cambridge, Downing Street, Cambridge, CB2 3DZ UK

**Keywords:** Buckwheat, Fagopyrum, Crop domestication, Agricultural origins, China, Polygonaceae

## Abstract

The two cultivated species of buckwheat, *Fagopyrum esculentum* (common buckwheat) and *F. tataricum* (Tartary buckwheat) are Chinese domesticates whose origins are usually thought to lie in upland southwestern China, outside the major centres of agricultural origins associated with rice and millet. Synthesis of the macro- and microfossil evidence for buckwheat cultivation in China found just 26 records across all time periods, of which the majority were pollen finds. There are few or no identifying criteria distinguishing *F. esculentum* and *F. tataricum* for any sample type. The earliest plausibly agricultural *Fagopyrum* occurs in northern China from the mid 6th millennium cal bp. The archaeobotanical record requires reconciliation with biogeographic and genetic inferences of a southwestern Chinese origin for buckwheat. Scrutiny of the genetic data indicates limitations related to sampling, molecular markers and analytical approaches. Common buckwheat may have been domesticated at the range margins of its wild progenitor before its cultivation expanded in the north, mediated by changing ranges of wild species during the Holocene and/or by cultural exchange or movement of early agriculturalists between southwest China, the Chengdu Plain and the southern Loess Plateau. Buckwheat probably became a pan-Eurasian crop by the 3rd millennium cal bp, with the pattern of finds suggesting a route of westward expansion via the southern Himalaya to the Caucasus and Europe.

## Introduction

The transition to agriculture in China occurred independently in at least three recognised centres (Zhao 2011). Dry land agriculture, with millets as the principal crops, began in the Loess Plateau and Yellow river catchment in north China. Rice agriculture developed in the middle and lower Yangtze valley and a third centre in tropical southern China, along the Zhujiang river south of the Nanling mountains, underwent an early agricultural transition in which roots and tubers, possibly including *Colocasia esculenta* (taro), were the main crops. The growth of Chinese archaeobotany in the last 10–15 years has rapidly advanced understanding of plant domestication in these regions and their interrelationships.

Buckwheat is an intriguing early Chinese crop whose origins appear not to fit the geography of any of these three recognised agricultural centres. In consequence, systematic evaluation of evidence for the origins of buckwheat has been neglected. Buckwheat is a pseudo-cereal belonging to the family Polygonaceae, with the grain either consumed whole after boiling or steaming, or ground into a gluten-free flour. Cultivated buckwheat comprises two species: *Fagopyrum esculentum* L. (common buckwheat) and *F. tataricum* Gaertn. (Tartary buckwheat). The two species differ in importance and cultivated range. *F. esculentum* is widespread in the temperate zones of the northern hemisphere (Ohnishi 1998b), while *F. tataricum* is principally a crop of high altitude zones, such as the circum-Himalaya region (Ohnishi 2000). Ecophysiologically, *F. tataricum* has some frost tolerance, which is lacking in *F. esculentum* (Campbell 1997). The two species also differ in breeding system. Tartary buckwheat is self-fertile and largely inbreeding (Tsuji and Ohnishi 2000), while *F. esculentum* is an insect-pollinated, obligate outbreeder (Cawoy et al. 2009).

The aim of this paper is to elucidate the geographical origins and early chronology of both *F. esculentum* and *tataricum* within China, through a synthesis of the archaeobotanical microfossil and macrofossil data, in the context of biogeography and genetic evidence. This project is timely for several reasons. First, although it has been concluded from biogeographic and genetic data that both species originated in southwestern China, specifically eastern Tibet, northern Yunnan and southwestern Sichuan (Konishi et al. 2005; Konishi and Ohnishi 2007; Ohnishi 2009), the agreement between the genetic and palaeobotanical data has never been examined. Second, the publication of palynological and macrofossil data from several new Chinese sites in recent years makes a review of the evidence for *Fagopyrum* appropriate. Third, the spread of agriculture into southwestern China and the Tibetan Himalaya region is a topic of much current interest (d’Alpoim Guedes 2011; d’Alpoim Guedes et al. 2013, 2014, 2015; Chen et al. 2015), to which an improved understanding of buckwheat origins and the differing ecologies of *F. esculentum* and *tataricum* is highly relevant. Finally, *F. esculentum* subsequently became a widespread crop in the Old World northern hemisphere, but the chronology of this globalization is uncertain (Jones et al. 2011; Boivin et al. 2012). A recent review of the European palynological and macrobotanical data (de Klerk et al. 2015) has highlighted this uncertainty. Here we undertake a comparable review of the data set for China. Understanding the spatial and temporal picture of buckwheat origins in China is an essential step to resolving its global pattern, including the status of buckwheat finds in Europe. These issues in turn relate to the wider topic of east–west crop spread.

## Methods

We aimed to collate all published data on archaeobotanical (comprising both macrofossil and microfossil) identifications of (buckwheat/*Fagopyrum*/qiaomai/荞麦) within the present boundaries of China. We searched the English and Chinese language literature using Google scholar and the China National Infrastructure Database (http://www.cnki.net) respectively, using various combinations of the search terms ‘*Fagopyrum*’, ‘buckwheat’, ‘vegetation’, ‘China’ on Google scholar and ‘qiaomai/荞麦’ (buckwheat) and ‘yizhi/遗址’ (archaeological site) on the China National Infrastructure Database. From each resulting record, we extracted site information, the number of finds of *Fagopyrum*, taxonomic identifications, and chronological information or dating results. We would emphasise that this is a meta-data survey; if a record has been published in a suitable medium, then it has been included. We have not gone back to either the original specimens or primary context sheets to further scrutinise those records.

To plot sites on a map, we used the longitudes and latitudes reported in the papers where this information was available, or estimated coordinates from other provided locality details using Google Earth. Maps were drawn using ArcMap v. 10.2, using imagery from NASA Blue Marble: Next Generation satellite imagery, originally produced by Reto Stockli and obtained from NASA’s Earth Observatory (NASA Goddard Space Flight Center); http://earthobservatory.nasa.gov/Features/BlueMarble/, and Adobe Photoshop CS4.

## Results

Twenty-six reports of *Fagopyrum* in the archaeological and/or palynological record in China were found (Table 1). Ten reports were of macrofossils, 14 of pollen records and two of starch granules.

**Table 1:**
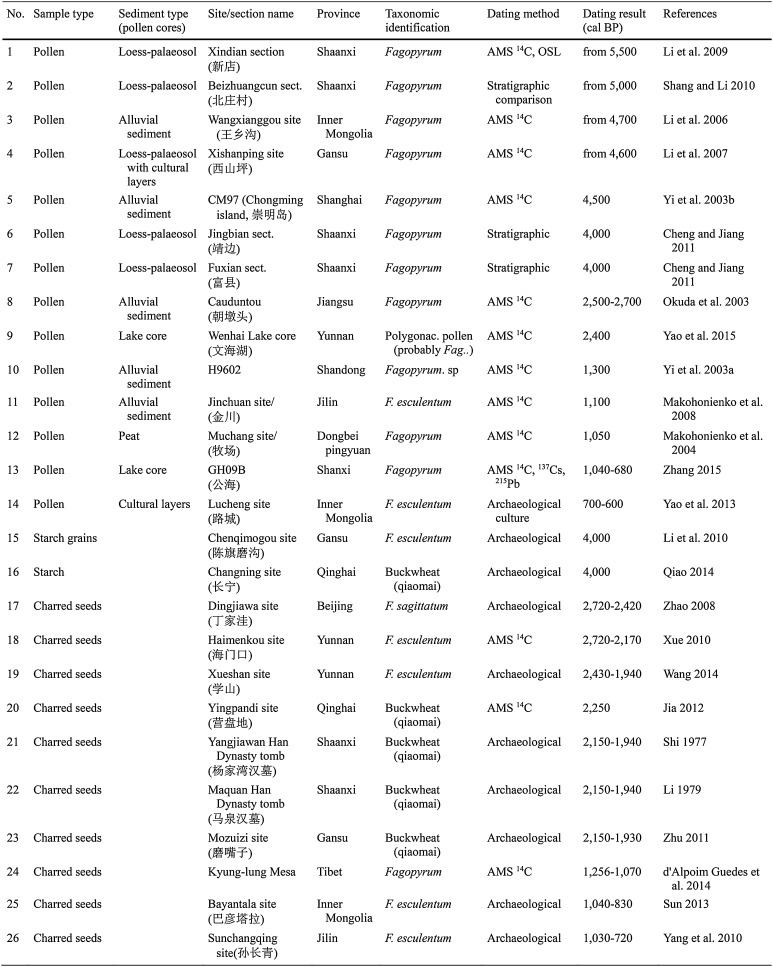
Sites and pollen sections in China with identifications of *Fagopyrum* (buckwheat)

It is important to emphasise the diversity of formation processes leading to the deposition of these different categories of fossils, not all of which are well understood. Probably the most straightforward in depositional terms are the pollen grains from confined lakes and peat accumulations, contexts which have been subject to much study and analysis. Given the limited release of *Fagopyrum* pollen to the wind, one possible interpretation of these records is as the result of crop processing activities in the immediate vicinity, although they may also relate, for example, to the deposition of whole flowers in the water or peat. In the case of unconfined waterways and active soils, it is more difficult to exclude either lateral or vertical movement of the contained pollen. In better studied crops, charred macrofossil deposition is most frequently associated with crop processing, and the same is likely to be true of buckwheat, though very much dependent on whether that processing took place near or far from domestic fires. In taphonomic terms, the mechanisms of persistence of starch granules in archaeological deposits are not well understood. In summary, there is a range of depositional processes with a substantial potential impact on the recovered data.

**Fig. 1:**
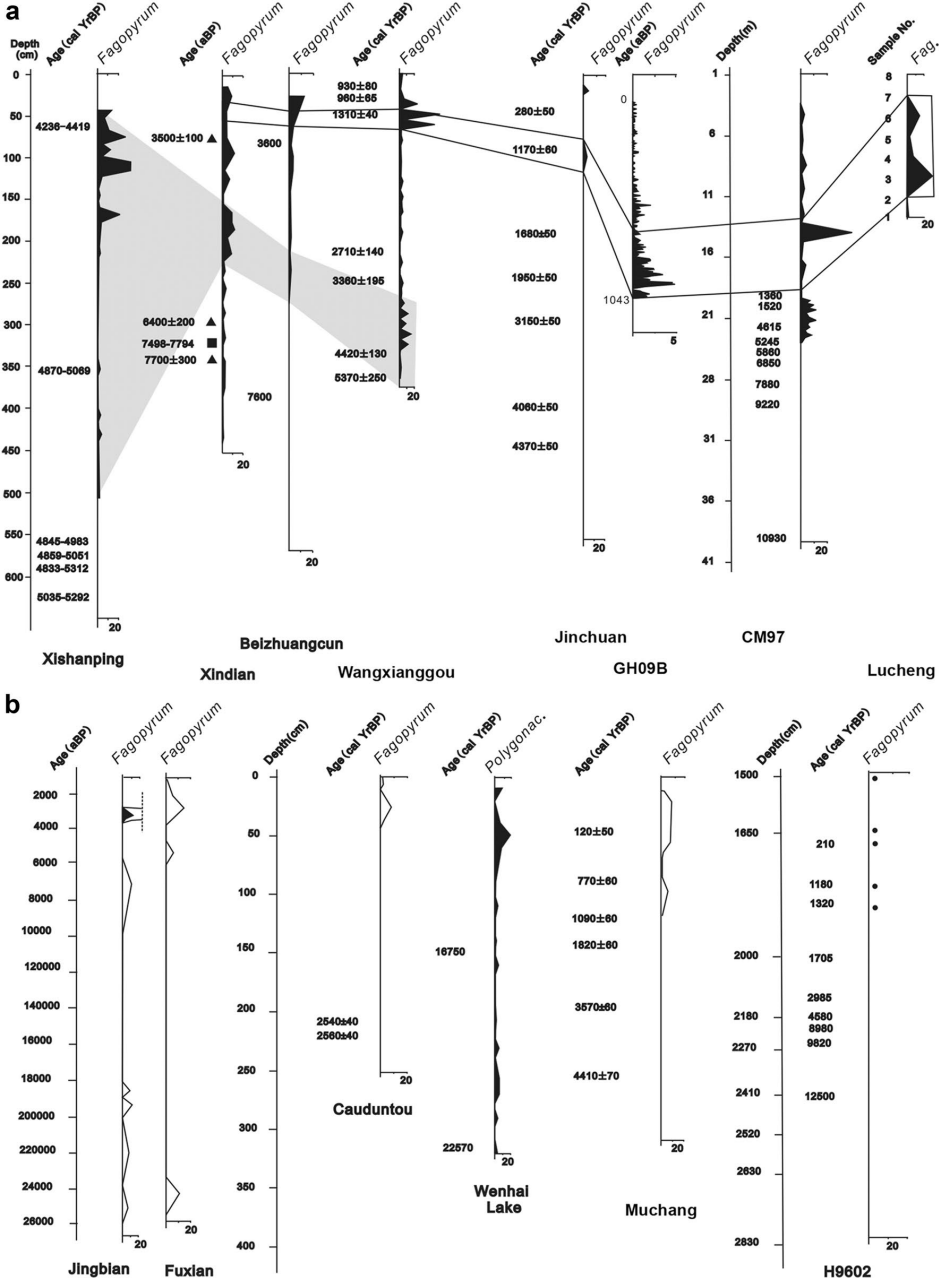
Diagrams of *Fagopyrum* pollen percentages in China. **a** Eight pollen diagrams with a relatively high amount of *Fagopyrum* pollen, which show two expansions of buckwheat in China during the Holocene (Li et al. 2006, 2007, 2009; Yi 2003b; Makohonienko et al. 2008; Shang and Li 2010; Zhang 2015; Yao et al. 2013). The grey shadow represents the first expansion of buckwheat during 5,000–4,000 bp; the hollow area represents the second expansion of buckwheat during 1,600–1,000 bp in China; **b** six pollen diagrams with low percentages of *Fagopyrum* pollen (Okuda et al. 2003; Yi et al. 2003a; Makohonienko et al. 2004; Cheng and Jiang 2011; Yao et al. 2015). Pollen diagrams reproduced from the references above with kind permission of the original authors, editors and publishers

**Fig. 2:**
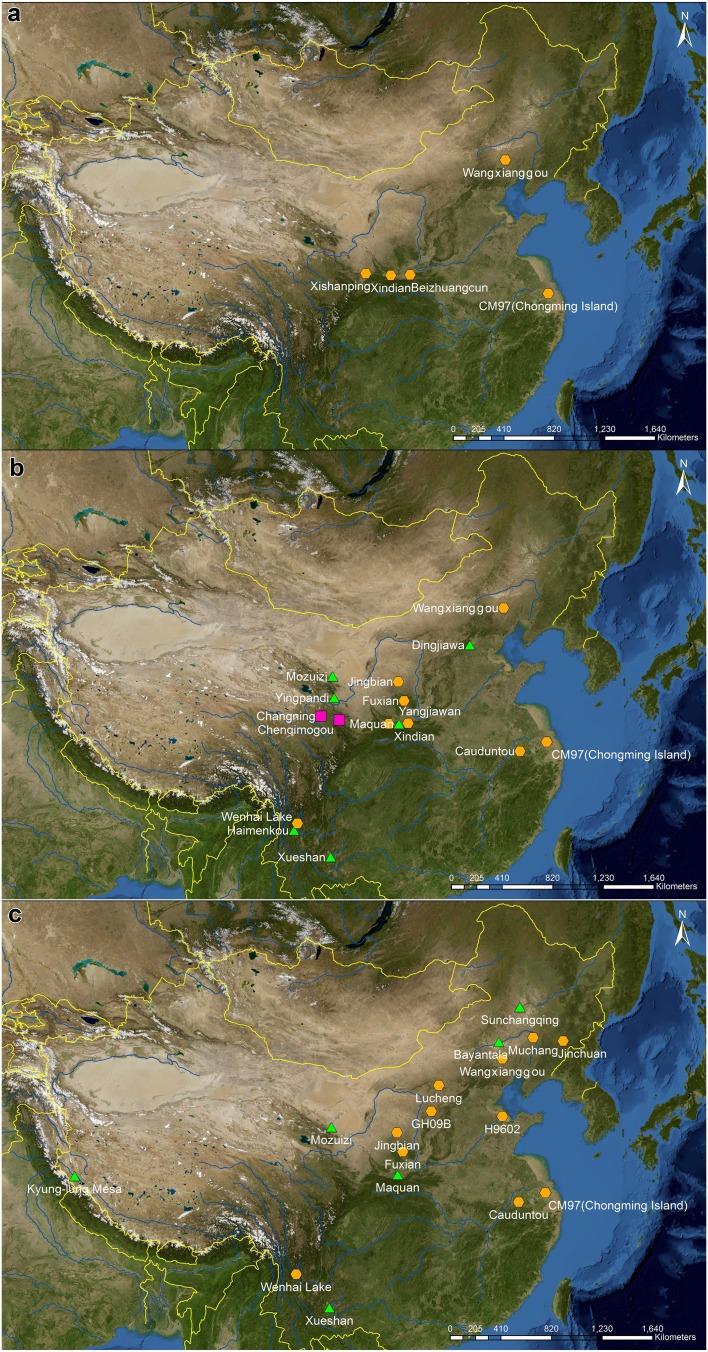
Location of sites and sections within modern-day China with identifications of *Fagopyrum* (buckwheat). **a** pre-4,000 cal bp; **b** 4,000–2,000 cal bp; **c** post-2,000 cal bp. Sample types: green filled triangle are charred seeds; red filled square are starch grains; orange filled hexagon are pollen sequence

For the pollen records, *Fagopyrum* or Polygonaceae pollen was sometimes present at a very low level along the entire depth of the core, as early as ~ 25,000 cal bp, as in the cases of Jingbian, Fuxian and Wenhai lake (Fig. 1). This great antiquity implies a wild form for at least the earlier part of the sequence, a point which is itself of interest. Taken at face value, this would suggest that a wild *Fagopyrum* species had a more extensive range in the past. This in turn could have implications for where in China domestication may have taken place (see the “Discussion”). For the purposes of this paper, we looked for suitable evidence for cultivation of *Fagopyrum*, and would propose that an abrupt increase in pollen count might be taken as a secure sign of human activity. We therefore took the date range for *Fagopyrum* pollen given in Table 1 and Fig. 2 to equate to the time from which it underwent a sharp percentage increase (Fig. 1), in line with the dates suggested by the original reports for evidence of *Fagopyrum* cultivation. Given this criterion, all records fall within the time period 5,500–700 cal bp.

Among the pollen cores or sections, ten use AMS ^14^C or OSL dating results, while three loess-palaeosol sections use stratigraphic cross-dating to estimate the date of samples, and the one section from an archaeological site is cross-dated by reference to material culture finds. These dating methods each have their own inherent constraints of precision and accuracy.

Eleven of the 14 pollen records are identified to genus level, as *Fagopyrum*. Identification criteria and taxonomic resolution of *Fagopyrum* pollen are discussed subsequently. While we comment on these, it was beyond the scope of this meta-survey to re-evaluate individual identifications, which would entail access to the original pollen samples.

The earliest abrupt increases in *Fagopyrum* pollen occur in the period 5,500–4,000 cal bp in a number of sites in northern China (Figs. 1, 2a). Four of these sites have direct sediment dating records: Wangxianggou (4,700 cal bp) in northeast China, Xindian (5,500 cal bp) and Xishanping (4,500 cal bp) in the northwest and Chongming (4,500 cal bp) at the mouth of the Yangtze river. At all these sites, *Fagopyrum* pollen is associated with an abundance of cereal pollen, seeds or charcoal. In the 4th and 3rd millennia cal bp, additional directly dated records with rises of *Fagopyrum* or Polygonaceae, probably *Fagopyrum*, pollen appear further upstream along the Yangtze at Cauduntou and in southwest China at Wenhai lake (Fig. 2b). A second major peak in *Fagopyrum* pollen occurred around 1,300–700 cal bp, comprising signals from a number of additional sites in the northeast (Fig. 2c) and a second abrupt increase around 1,100 cal bp in the Wangxianggou site. At two of these sites, Jinchuan and Lucheng, pollen is specifically identified as *F. esculentum*, but no criteria or justification are given.

The records above include pollen from confined lakes and peat accumulations (five sites); unconfined waterways (three sites) and active soils and sediments (six sites). Similar trends are observed in each of these groups. As indicated above, the confined lakes and peat accumulations, which are the most secure types of deposits, display this pattern, and it is echoed in the depositional contexts that may be open to greater taphonomic complexity.

Contemporary with the latest of the first set of *Fagopyrum* pollen rises, culturally dated to around 4,000 cal bp, are two published records of starch grains from northwestern China. At Chenqimogou, five starch grains identified as *F. esculentum* were present among a total of 48 from human dental calculus, although the number of samples was very small. At Changning, nine *qiaomai* (buckwheat) starch grains were among 152 reported from stone knives.

*Fagopyrum* macrofossils were found in the form of charred seeds at all ten sites where they have been reported; these records are mostly limited by few samples and/or lack of any detailed information. Identifications are to species level as *F. esculentum* (including the synonym *F. sagittatum*) or the vernacular *qiaomai*, with the exception of the record from Kyung-lung Mesa in southwestern Tibet, identified as *Fagopyrum* sp. and inferred to be a wild species on account of the small size (3 mm) of the preserved nutlets. Seven records are from the 3rd millennium cal bp, spanning the Zhou dynasty, Warring States period and Han dynasty, and have a wide geographical distribution in northeastern, northwestern and southwestern China. At Haimenkou in Yunnan province, the earliest site with radiocarbon dating (3,050–2,750 cal bp), only three grains of *F. esculentum* were found, but the nearby site of Xueshan, dated culturally to the end of the 3rd millennium bp, had 149 grains. At the three Han dynasty sites in the Yellow river/Loess Plateau region, buckwheat was associated with pottery in tombs, but these excavations were carried out in the 1970s and minimal archaeobotanical detail was recorded.

Four grains of *F. esculentum* were recovered from each of two Liao dynasty sites in northeastern China (Bayantala and Sunchangqing). These coincide geographically and chronologically with the second major set of *Fagopyrum* pollen rises, around 1,000-700 cal bp. The northeastern focus of buckwheat finds later than 2,000 cal bp resonates with its modern importance, as inferred from the number of buckwheat accessions in the Chinese Crop Germplasm Information System (CGRIS). A geographical outlier of similar period is the macrofossil record of possibly wild *Fagopyrum* sp. from the Zhangzhung kingdom site of Kyung-lung Mesa in southwestern Tibet.

There have been no positive identifications of *F. tataricum* from any sample type of any period in China.

## Discussion

### Interpreting the *Fagopyrum* pollen and macrofossil record

The majority of records were identified to genus level as *Fagopyrum*. Chinese palynological work predominantly uses the reference criteria of Wang et al. (1995); Zhou et al. (2003) and Chen et al. (2014). These authors consider that *Fagopyrum* pollen cannot be identified to species based on size, shape or surface ornamentation. Zhou et al. (2003) describe the pollen morphology of the genus *Fagopyrum* Mill. in China as ‘prolate, or often subprolate/prolate to spheroidal in shape, and elliptical from equatorial view, circular from polar view, with their germination aperture being all 3-colporate’. Wang et al. (1995) agree that *Fagopyrum* species produce prolate shaped, tricolpate pollen with reticulate ornamentation. Palynologists from Europe also describe *Fagopyrum* pollen as tricolporate, oval with branched columellae, with variable sizes from 40 to 60 μm in glycerine; sizes are dimorphic in heterostylous species including *F. esculentum* (Fægri and Iversen 1989; Moore et al. 1991; de Klerk et al. 2015). However, work on *Fagopyrum* pollen in Europe has more often attempted to distinguish *F. esculentum* and *F. tataricum*, following the identification criteria of van Leeuven et al. 1988, who consider that the two species are distinguishable on the base of basal trunks which are either distinct (*F. esculentum*—branched columellae in the mesocolpium as well as at the apocolpium and very thick exine) or indistinct (*F. tataricum*). For the two sites in China (Jinchuan and Lucheng) where pollen identifications are reported to species level as *F. esculentum*, insufficient detail or explanation of identification criteria is given to infer whether this taxonomic precision is appropriate.

It has been claimed that the pollen of a number of African genera of Polygonaceae (*Oxygonum, Antigon* and *Afrobrunnichia*) is morphologically similar to that of *Fagopyrum* (de Klerk et al. 2015), but the basis of the claim is unclear and African genera are in any case unlikely to be relevant to the topic of this paper. Chinese pollen morphology studies show that *Fagopyrum* pollen has surface sculpture distinguishing it from other genera, which allows confident genus level identification in sedimentary pollen diagrams (Wang et al. 1995).

**Fig. 3:**
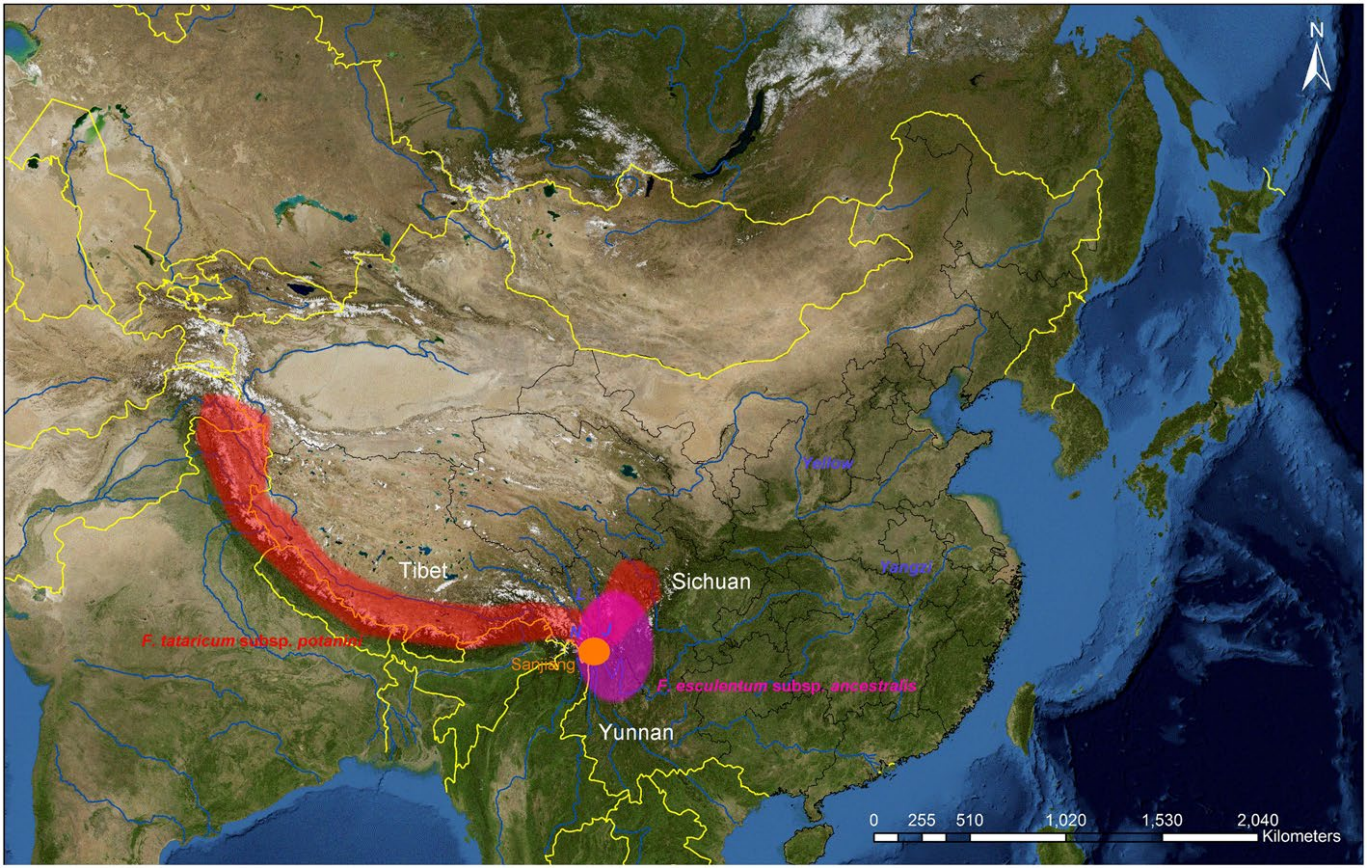
Approximate distribution of the wild subspecies *F. esculentum* ssp. *ancestralis* and *F. tataricum* ssp. *potanini*, in relation to Chinese provinces and rivers mentioned in the text. The Sanjiang area inferred by Konishi et al. (2005), Konishi and Ohnishi (2007) and Ohnishi (2009) as the centre of origin of domesticated *F. esculentum* is also shown. Rivers in the Sanjiang area shown as initials on the map *J* Jinsha, *L* Lancang, *N* Nu. Based on Ohnishi (1998a) and Tsuji et al. (1999)

Seed and pollen records of *Fagopyrum* in China outside the south-west are generally assumed to represent cultivation of one of the two domesticated taxa, even in the absence of species identification (Fuller et al. 2018). Two lines of reasoning lead to this assumption. First, most wild *Fagopyrum* species, including *F. esculentum* ssp. *ancestralis* and *F. tataricum* ssp. *potanini*, are restricted to southwestern China (Fig. 3), although it should be noted that perennial buckwheat (*F. dibotrys*) is more widespread across southern China and as far north as Henan, Shaanxi and southern Gansu provinces (Yamane et al. 2003; http://www.efloras.org/florataxon.aspx?flora_id=2&taxon_id=242100052); the implications of this are discussed below. Second, by reference to associated taxa, *Fagopyrum* pollen always appears within an open landscape or forest clearance episodes. *Fagopyrum* pollen is invariably encountered in low numbers, on account of its being entomophilous or self-pollinated, with poor production and limited dispersal capacities on account of the large pollen grain size, as well as the coarse sculpture of the exine (Miras 2009; Pidek 2009; de Klerk et al. 2015). The levels of *Fagopyrum* pollen in surface samples, both in situ and near contemporary buckwheat fields are markedly low (Miras 2009; Pidek 2009; de Klerk et al. 2015). We therefore tentatively suggest that a high incidence of buckwheat pollen might be explained by crop processing in the vicinity.

Trigonous nutlets are not uncommon in carbonised macrofossil assemblages, and are typically attributable to one of two families, Cyperaceae and Polygonaceae, on the basis of surface patterning and terminal attachments. Within the Polygonaceae, details of these features along with cross section, sharpness of lateral ridges and overall size normally allow further attribution to genus (Cappers et al. 2006). The large size and surface texture of *Fagopyrum* nutlets are distinctive, and published records are unlikely to be problematic. Within the genus, species identification may be suggested on the basis of size, as *F. tataricum* is on average rather shorter than *F. esculentum*, though there is some overlap in their ranges. Their pericarps have structures distinctive to particular species, which may also be discerned in surface patterning (Winton and Winton 1932).

The small number of buckwheat macrofossil records from China is striking. As indicated above, in better studied crop taxa, the incidence of charred macrofossils is related to crop processing activities in the vicinity of routine fires, generally domestic fires of some kind. Not all ethnographically attested crop processing occurs near fire; indeed some processes are actively kept distant from fires, in which case they leave no charred record. It is thus perfectly possible, if conjectural, that a low incidence of macrofossils relates to the distance of crop processing activities from fires, rather than any underlying level of incidence in the agrarian landscape.

There is also an increasing interest in starch granules, with some attributions to *Fagopyrum* (Li et al. 2010; Qiao 2014). Both the criteria for genus and species identification and the circumstances that might allow their persistence for thousands of years are matters for ongoing investigation.

### Biogeography and genetic evidence for the origins of cultivated buckwheat

The geography of cultivated buckwheat origins has been inferred primarily from biogeographic and genetic data. China is the centre of species diversity of *Fagopyrum*, which comprises around 16 species, many of which have a restricted endemic distribution in southwestern China (Ohnishi and Yasui 1998; Chauhan et al. 2010). The taxa characterised in recent years include the wild progenitors of both common and Tartary buckwheat. Wild *F. esculentum*, designated *F. esculentum* ssp. *ancestralis*, resembles cultivated common buckwheat morphologically and as a self-infertile, heterostylous, outcrossing diploid taxon with 2n = 16 chromosomes, but differs in its smaller flowers and achenes, thicker leaf blades, strong seed dormancy, shattering of premature achenes, a more branching habit at lower nodes and a longer vegetative growth period (Ohnishi 1991, 1998a). Its known distribution is limited to a ~ 250 km radius in northern Yunnan province, southwestern Sichuan province and eastern Tibet, where it typically grows on rocky cliffs and roadsides (Fig. 3; Ohnishi 1998a, b, 2009; Ohnishi and Yasui 1998; Ohnishi and Konishi 2001; Ohnishi and Tomiyoshi 2005). Its progenitor status was inferred from the typical wild species traits above, and the resolution of ssp. *esculentum* and ssp. *ancestralis* as sister taxa within phylogenies of *Fagopyrum* was based on morphological traits, isozyme variability and RFLPs of cpDNA (Ohnishi and Matsuoka 1996).

Within the narrow distribution of *F. esculentum* ssp. *ancestralis*, the Sanjiang area along the Tibet–Sichuan border, where three major rivers, the Changjiang, Mekong and Salween, flow north–south in deep valleys between 3,000–4,000 m high mountain ranges, has been inferred to be the geographic origin of domesticated *F. esculentum* (Fig. 3). This suggestion follows from genetic analyses of wild populations and domesticated landraces in the same geographical region, using amplified fragment length polymorphism (AFLP), simple sequence repeat (SSR) and isozyme markers, based on the monophyly of the cultivated populations and their close relationship to wild populations from this area (Konishi et al. 2005; Konishi and Ohnishi 2007; Ohnishi 2009).

A wild subspecies of Tartary buckwheat, *F. tataricum* ssp. *potanini* Batalin, has a relatively wide distribution in northern Sichuan province, southern Tibet, Kashmir and northern Pakistan and more sporadically in Gansu and Qinghai provinces (Ohnishi 1993a, 1994, 1998a, b, Fig. 3). In Tibet it reaches altitudes of up to 4,900 m a.s.l. (Ohnishi 1993a). Like cultivated Tartary buckwheat, ssp. *potanini* is a self-fertile, homostylous, inbreeding diploid with 2n = 16 chromosomes. The literature describing its morphology is difficult to access. Weedy types of Tartary buckwheat also exist, distributed in northern Pakistan, and are described as morphologically similar to cultivated landraces but with characters like the wild subspecies, including a shattering seed head habit, strong dormancy, and much branching (Ohnishi 1994).

*Fagopyrum tataricum* (ssp. *tataricum*) and *F. tataricum* ssp. *potanini* were confirmed as sister taxa (Ohnishi and Matsuoka 1996), from which the authors inferred *F. tataricum* ssp. *potanini* to be the wild ancestor of cultivated Tartary buckwheat. Isozyme, RAPD and AFLP variability was found to be substantially higher in wild than in cultivated or weedy Tartary buckwheats (Ohnishi 1998b; Tsuji and Ohnishi 2000, 2001), and the authors inferred that the cultivated subspecies was domesticated in eastern Tibet/Yunnan/Sichuan provinces on the basis of the high genetic diversity in wild populations from this area, despite the genetic similarity of cultivated landraces to wild populations from Tibet and Pakistan (Ohnishi 1998b; Tsuji and Ohnishi 2000, 2001). Weedy *F. tataricum* was dispersed among wild and cultivated groups in the RAPD phylogenetic analyses and it was suggested that these forms arose from hybridization between wild and cultivated plants in Yunnan or Sichuan, which later spread to northern Pakistan as weeds of cultivated *F. tataricum*.

### Limitations of the genetic data

The inference of the Sanjiang region as the centre of origin of cultivated *F. esculentum* (Konishi et al. 2005; Konishi and Ohnishi 2007; Ohnishi 2009) may constitute over-interpretation of the limited genetic data to date. Very few samples, particularly of cultivated *F. esculentum*, were included in these genetic studies. As the authors admit, their failure to sample the cultivated taxon from outside southwest China is a serious weakness, which limits the robustness of inferences of the relationship between ssp. *ancestralis* and ssp. *esculentum*. The genetic markers used are now outdated, with particular weaknesses being the low level of variability detected by isozymes and problems of dominance and false monophyly associated with AFLPs (Allaby and Brown 2003). We also suggest that the data in Ohnishi (2009) offer inadequate support for an origin in Sanjiang, as this interpretation relies on the weak statistic of genetic distance measures and moreover virtually all the wild populations analysed were genetically close to the cultivated samples. Given the geographical range of *F. esculentum* ssp. *ancestralis*, the origin of *F. esculentum* ssp. *esculentum* somewhere in the eastern Tibet/northern Yunnan/western Sichuan region seems uncontroversial, but more precise definition is premature.

The centre of origin of Tartary buckwheat is in principle less geographically constrained, given the wide range of *F. tataricum* ssp. *potanini*. However there is a lack of any specific archaeobotanical records of *F. tataricum* in any period, considering the general shortage of records discussed above, a situation that could be mitigated by further research. The argument that the domestication of *F. esculentum* in eastern Tibet/Yunnan/Sichuan supports a domestication of *F. tataricum* in the same region (Ohnishi 1998b; Tsuji and Ohnishi 2000, 2001) is constrained by the lack of associated archaeological evidence for the beginnings of cultivation. The actual genetic evidence for domestication in this region relies solely on maximum genetic diversity, but this inference is made on the basis of very few variable loci, which was a frequent problem of first-generation molecular markers. It is also notable that *F. tataricum* landraces are phylogenetically closer to wild populations in central Tibet and Pakistan. Tsuji and Ohnishi (2000) speculate that these morphologically wild populations are descended from hybrids between cultivated and wild populations in Yunnan or Sichuan, but their data do not directly support this hypothesis.

### Buckwheat domestication and cultivation in China: where and when?

Based on the above evidence, the direct sediment dating records associated with *Fagopyrum* pollen, together with the abundance of cereal pollen, seeds and charcoal, are consistent with buckwheat cultivation having arisen in northern China from around 5,500 bp. This is outside the inferred centres of domestication of both common and Tartary buckwheat in eastern Tibet/Yunnan/Sichuan, where the genus does not appear in the archaeobotanical record of pollen or macroremains until the 3rd millennium cal bp. This lack of agreement between the archaeobotanical and the genetic evidence demands further attention, and has hitherto been underplayed: d’Alpoim Guedes et al. (2014) state that the palyonological evidence for buckwheat from northeastern and northwestern China and the lower Yangtze ‘postdates 2000 bc’, citing Boivin et al. (2012); however, the latter paper actually mentions dates from 2400 to 2500 bc, and omits mention of the earlier records from Xindian and Beizhuangcun.

The possible scenarios that explain buckwheat origins in the light of these differing lines of evidence are necessarily speculative, given the very limited state of knowledge about domestication traits, role in subsistence and taxonomic specificity in cultivated *Fagopyrum*. Nevertheless, they highlight some important avenues for further investigation.

First, the possibility that buckwheat (particularly *F. esculentum*) underwent an initial domestication from *F. esculentum* ssp. *ancestralis* at the margins of the latter’s range in southwestern China, but was not substantially cultivated until it spread beyond that range into the north, is interesting in relation to the obligate outbreeding nature of *F. esculentum*. Reproductive isolation of outcrossing crops from their wild progenitors, a keystone of both morphological and phylogenetic concepts of domestication, is expected to be slower and/or more complicated compared with taxa which self fertilise. The empirical evidence gives some support to this expectation, although it is difficult to disentangle the effect from associated traits, in particular annuality (Glémin and Bataillon 2009). Although geographical isolation of domesticated crops from their wild progenitors appears to be the exception rather than the norm (Dempewolf et al. 2012), we can postulate that northwards ‘translocation’ in the loosest sense (see below) of *F. esculentum* populations characterised by some domestication traits facilitated fixation of these traits to make a sufficiently productive crop to reach detectable levels in the archaeobotanical record.

**Fig. 4:**
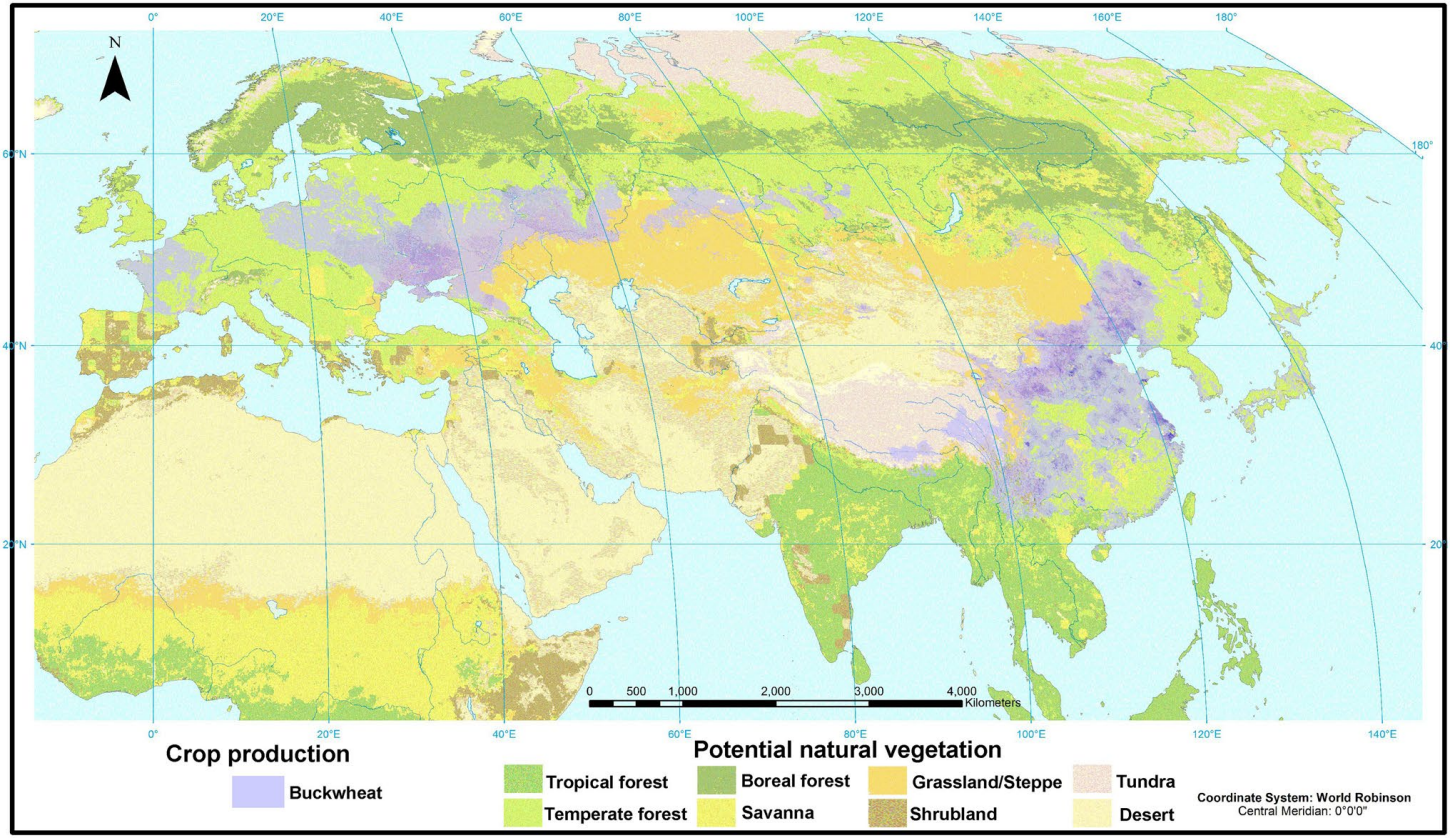
Distribution of buckwheat cultivation in relation to potential natural vegetation. Buckwheat cultivation data represent total crop production in metric tons; darker colour represents higher production. Data from http://www.earthstat.org/data-download/ (last accessed 03 April 2017), based on data in Monfreda et al. (2008), which contains full data description. Potential natural vegetation data from http://www.earthstat.org/data-download/ (last accessed 03 April 2017), based on Ramankutty and Foley (1999) which contains full data description

This hypothesis would demand explanation of the mechanism of geographical isolation or translocation. One obvious possibility is that the range of *F. esculentum* ssp. *ancestralis* extended into northern China in the mid-Holocene. Palynological vegetation reconstructions indicate that temperate forests extended further north in China than they do today, including around the Xindian site on the southern Loess Plateau (Shang and Li 2010; Ni et al. 2014). The range of *F. esculentum* ssp. *ancestralis* in Yunnan, Sichuan and eastern Tibet today appears also to fall broadly within a forest rather than grassland biome, providing some support for this idea, but much further work is needed on the precise ecological niche of the wild taxon and the abiotic and biotic factors that may govern this. As a related issue, cultivated buckwheat today predominates in steppe or forest-steppe zones (Fig. 4), suggesting the interesting possibility of a shift in ecological adaptation following domestication.

A second possibility for northwards movement of common buckwheat in China is small-scale cultivation and localised domestication in the southwest followed by translocation by people to central and northern China. The development of agriculture in the Chengdu Plain and southwest China are a topic of increasing interest in Chinese archaeology (d’Alpoim Guedes 2011; d’Alpoim Guedes et al. 2013, 2014, 2015), although virtually always from the perspective of south and westward movement of millet and rice agriculture and agriculturalists. Evidence of cultivation of *Setaria* and *Panicum* (foxtail and broomcorn millets) appears from around 5,500 cal bp at the Neolithic sites of Changdu Karuo in eastern Tibet and Haxiu and Yingpanshan in northern Sichuan (d’Alpoim Guedes 2011). All these sites lie further north, by around 500 km, than the region of origin of domesticated *F. esculentum* proposed by Ohnishi and colleagues. In Yunnan province, the earliest evidence for agriculture comes from rice remains and may date from around 4,500 cal bp at Haidong in eastern Yunnan and Baiyangcun, although systematic flotation and reliable dating only comes from around 3,600 cal bp at Haimenkou, where buckwheat is also present in levels dated around 3,050–2,750 cal bp. A recent analysis of site chronology from the Nujiang valley in northwestern Yunnan, the area inferred from biogeography and genetics as the centre of origin of domesticated *F. esculentum*, found convincing evidence for Neolithic settlement of the region only from ~ 4,200 cal bp. Earlier dates (~ 5,100 cal bp) exist from the first stage of Haimenkou, but may not be reliable (Liu et al. 2016). Prior to this period, there is evidence for occupation of a handful of late Palaeolithic sites in the region by hunter-gatherers, such as Tangzigou (~ 8,000 cal bp; Liu et al. 2016). In summary, unless these hunter-gatherer populations were independently experimenting with buckwheat cultivation and sustained this until cultural exchange with northern China began in the 6th millennium cal bp, the currently understood chronology of Neolithic and/or agricultural sites around the Yunnan/Sichuan/Tibet border postdates apparent buckwheat cultivation in the north by as much as 1,000 years. However, given the clear cultural links between central Sichuan and northern China by the mid 6th millennium cal bp, we could speculate regarding a common buckwheat domestication in Sichuan and its northward expansion ‘contraflow’ to that of millet, perhaps through the Majiayao and Yangshao cultures. This scenario requires that the past range of *F. esculentum* ssp. *ancestralis* extended further to the north than today. Although any of these scenarios demands much substantiation, resolving the questions around the domestication and expansion of buckwheat within China is of interest precisely because its geography is atypical of other elements of the agricultural package, and may well add complexity to the emerging narrative of Neolithic cultural interactions.

### Buckwheat as a Tibetan plateau/Himalayan crop

The expansion of cultivated buckwheats, both *F. esculentum* and *F. tataricum*, is also of interest in relation to the arrival of agriculture on the Tibetan plateau. Both common and Tartary buckwheat and their respective wild ancestors are native to the low mountain zones at the southeastern edge of the region.

Research on agricultural spread to the Tibetan Plateau has focused chiefly on the major crops found in archaeobotanical assemblages from the region, which are wheat, barley and broomcorn and foxtail millets. Their potential ranges have also been assessed using ecological modelling (d’Alpoim Guedes et al. 2014, 2015; Chen et al. 2015). The potential limits of cultivation of common and Tartary buckwheat have not yet been comparably modelled, and beyond the sites of Haimenkou, Xueshan and Wenhai lake at the southeastern margins of the Tibetan Plateau, no sites in the eastern part of the plateau have yielded *Fagopyrum* remains. However, the majority of archaeobotanical investigations have been on the northeastern plateau (Chen et al. 2015), and more research is needed on sites in its southeast. Some 1,000–1,500 km to the west, to the south of the Tibetan Plateau in the Himalaya, *Fagopyrum* remains, both *F. esculentum* and *F. tataricum*, appear in western central Nepal from 3,000 cal bp in the Jhong river valley at 3,000–4,000 m a.s.l. (Knörzer 2000), around 1,000 cal bp at Kohla at 3,350 m, a single grain of *Fagopyrum* cf. *esculentum*, and in southwestern Tibet small nutlets, possibly of a wild species, around 700–880 ad at Kyung-lung Mesa (d’Alpoim Guedes et al. 2014). It has been suggested that the cereal remains were brought to these sites from lower altitudes rather than being cultivated in situ. From the very limited data available, we can postulate that domesticated *F. esculentum* spread westwards along the southern slopes of the Himalaya by the 3rd millennium cal bp; whether *F. tataricum* followed a similar expansion or was a local Himalayan domesticate remains an open question. From the current distribution of *F. esculentum* and *F. tataricum*, Ohnishi (1993a) concludes that buckwheat did not cross the Himalayas and reach into the central Tibetan Plateau.

### Spread into western Eurasia

The arrival of cultivated and Tartary buckwheat in Europe is widely considered to date back only to the late medieval period, as summarised by de Klerk et al. (2015). However, the presence of earlier *Fagopyrum* pollen records in Europe prompted Janik (2002), Jones (2004) and Jones et al. (2011) to challenge this narrative, raising the possibility that cultivated buckwheat spread into Europe as early as the 7th millennium cal bp. To explore this hypothesis further, de Klerk et al. (2015) assembled a comprehensive data set of pre-medieval European records, identifying some 232 pollen and ten macrofossil records attributable to *Fagopyrum* dating prior to 650 cal bp. The earliest time period with layers containing *Fagopyrum* pollen is before 11,700 cal bp, suggesting that a non-agricultural explanation is required in any case for at least some of these pre-medieval finds. Many of the sites collated in this survey contained just a single pollen grain attributed to *Fagopyrum*, and an assessment of indicators of cultivation using criteria comparable to this paper was not made. de Klerk et al. (2015) suggest that pollen records prior to 4,000 cal bp could represent a wild *Fagopyrum* or related Polygonaceae taxa now extinct in Europe. The few Bronze Age and earlier macrofossil records in Europe require further scrutiny.

In the period between 4,000 and 2,800 cal bp, many of the samples containing buckwheat come from the Bronze Age (3,500-3,400 cal bp) Georgian cemetery of Saphar-Kharaba (Kvavadze 2007). Interestingly, this chronology in the Caucasus is comparable to new data on the earliest firm evidence for broomcorn and foxtail millet in the region (Lucie Martin and Nana Rusishvili, personal communication), cereals with some ecological similarities to buckwheat whose spread from China to Europe has sometimes been considered together (Jones 2004; Jones et al. 2011). In contrast to the Asian millets, however, for which the macrofossil picture in the central Asian Bronze Age has been clarified considerably by recent work (Spengler and Willcox 2013; Spengler et al. 2013, 2014a, b, c), no *Fagopyrum* macrofossil records from central Asia have emerged. It is also notable that buckwheat is absent from the diverse range of excellently-preserved crops at Bronze Age cemetery sites in Xinjiang in the far northwest of China, in which broomcorn and foxtail millet typically featured prominently (Jiang et al. 2007, 2013; Jia et al. 2011; Li et al. 2013).

### The pattern and drivers of buckwheat globalization

In summary, although the initial zone of buckwheat domestication requires clarification, the archaeobotanical data indicate its cultivation in northern China from at least the mid 6th millennium cal bp, and in southwestern China and the Tibetan Plateau/Himalaya from at least the end of the 4th millennium cal bp. It may also have been cultivated in the Caucasus from the 4th millennium cal bp. de Klerk et al. (2015) conclude from the increase in European pollen and macrofossil records that buckwheat cultivation in Europe was very probably already widespread by the 3rd millennium cal bp, and possibly from 3,800 cal bp. The absence of central Asian records of buckwheat, together with these positive identifications in the Himalayan and Caucasus regions in the 4th–3rd millennia cal bp, could suggest that buckwheat spread to Europe via a southerly route. This would indicate a westward expansion separate from that of broomcorn millet; the archaeobotanical data for foxtail millet have rarely been considered independently. Most authors suggest that the eastward spread of buckwheat to Japan occurred from around 4,000 cal bp (de Klerk et al. 2015, Fuller et al. 2018); the significance of the earlier (5,500 cal bp) buckwheat pollen record of Tsukada et al. (1986), as with similarly early *Fagopyrum* pollen finds in Europe, is hard to interpret, with possibilities comprehensively discussed by de Klerk et al. (2015). Genetic data addressing the route of spread of buckwheat are very limited (Ohnishi 1993b, c; Murai and Ohnishi 1996) and appear to give conflicting answers. Further genetic and palynological and/or archaeobotanical work is needed to clarify the routes of spread and whether the geographical ‘gap’ in Russia and central Asia or around the Caspian region can be bridged.

Returning to the narrative of ‘arrival’ of common buckwheat in Europe in the late medieval period, we can hypothesise that its increased presence in the macrofossil record from this period may instead relate to an episode of intensification of cultivation. It would be interesting to seek parallels between this and the possible earlier intensification in northern China. The dynamics of intensification of common buckwheat cultivation could be peculiar to its biology as an insect-pollinated crop. Specifically, modern data show that honeybee pollination substantially increases buckwheat yield (Klein et al. 2007). This raises the interesting possibility that human management of bee populations was among the drivers of buckwheat globalization.
